# Mesenchymal stem cells in osteotomy repair after tibial tuberosity advancement in dogs with cranial cruciate ligament injury

**DOI:** 10.1186/s40634-018-0130-z

**Published:** 2018-06-14

**Authors:** Clarissa Rocha dos Santos, Richard da Rocha Filgueiras, Patrícia Furtado Malard, Andre Rodrigues da Cunha Barreto-Vianna, Kaique Nogueira, Carolina da Silva Leite, Eduardo Maurício Mendes de Lima

**Affiliations:** 1 Faculty of Agronomy and Veterinary Medicine, University of Brasilia, Brasília, DF Brazil; 2 Orthos Ortopedia e Neurocirurgia Veterinária, Brasília, DF Brazil; 3 BIO-Biotecnologia em Reprodução Animal, Brasília, DF Brazil; 4 Faculty of Veterinary Medicine, Federal University of Lavras, Lavras, MG Brazil

**Keywords:** Orthopedics, Biotechnology, Joints, Knee

## Abstract

**Background:**

The cranial cruciate ligament rupture (CCLR) is the most commonly encountered orthopedic condition in dogs. Among the various techniques to treat this condition, tibial tuberosity advancement (TTA) has been used to obtain rapid recovery of the affected knee. The objective of this study was to evaluate the viability of the use of mesenchymal stem cells (MSC) implanted in the osteotomy site obtained by TTA in nine dogs diagnosed with CCLR.

**Methods:**

The MSC were isolated from the adipose tissue of the dogs and cultured for eight days, the animals were divided into two groups. Animals from the treated group (GT) received cell transport medium containing about 1.5 millions MSC, and the animals from the control group (GC) received only the cell transport medium. The study was performed in a double-blind manner using radiographs acquired on days 15, 30, 60 and 120 after the procedure. Evaluations of the density of the trabecular bone were performed using image analysis software. The results were subjected to descriptive statistical analysis, followed by the normality test, Chi-square test, Mann-Whitney test and Tukey’s multiple comparison test for *p* ≤ 0.05.

**Results:**

After 30 days of the procedure, the animals of the GT presented an ossification mean 36.45% greater (*p* ≤ 0.033) than the GC, and there were no statistical differences for the other periods.

**Conclusions:**

Despite the total bone ossification within the expected period, there was no minimization of the estimated recovery time with the application of MSC, and inflammatory factors should be considered for reassessment of the therapeutic intervention time.

## Background

The main function of the cranial cruciate ligament is the stabilization of the knee, where it prevents cranial displacement of the tibia in relation to the femur, in addition to aiding the rotational movements of the tibia (Calvo et al. 2014; Fossum 2015; Venzin et al. 2004). Cranial cruciate ligament rupture (CCLR) is the orthopedic knee condition most common in dogs (Calvo et al. 2014; Marino and Loughin 2010). The causes that lead to CCLR are still not completely defined (Vedrine et al. 2013), but a number of presumed factors lead to a common pathway in the origin of these lesions (Cook 2010). Although the lesions are primarily traumatic, other degenerative factors have been noted in the pathogenesis of this condition, such as age, sex, and obesity (Venzin et al. 2004), as well as racial predisposition, inflammatory and conformation alterations, and immune-mediated mechanisms (Griffon 2010).

The extracapsular techniques usually applied for the treatment of this condition are the fabelo-tibial suture, osteotomy of the tibial plateau, and advancement of the tibial tuberosity (TTA). In addition, there are intracapsular techniques that most commonly use the autogenous fascia lata as material. Synthetic materials have not been widely used due to the possibility of distension, rupture, or infection (Fossum 2015).

The TTA demonstrates excellent results due to lack of alteration of the original geometry of the knee joint and its promoting of the distribution of pressure in the cartilage (Hoffmann et al. 2006). In addition, this technique is a simple surgical procedure with less potential to generate adverse effects due to technical problems (Boudrieau 2009). The use of the TTA technique in combination with the application of stem cells provides a new option for the treatment of CCLR.

Bone formation and repair are performed by osteoprogenitor cells derived from mesenchymal stem cells (MSC) that act in the lesion area by signaling cytokines that stimulate the replication and differentiation in active osteoblasts. The osteoblasts deposit an extracellular matrix mineralized with hydroxyapatite (Kraus and KIRKER-HEAD 2006). In addition, MSC also maintain viability and potential multi-lineage after cryopreservation, so MSC can be effectively isolated, expanded, preserved and implanted (Kraus and KIRKER-HEAD 2006).

Kang et al. (2012) found that MSCs from adipose tissue have a higher proliferation potential than the other types of MSCs and that their main advantages over other types of MSCs include easy availability in substantial quantities and that they can be collected without risk to the donor (Schäffler and Büchler 2007).

The aim of this study was to analyze the feasibility of using MSCs implanted at the osteotomy site obtained during the TTA technique in dogs with a CCLR diagnosis and to verify the clinical recovery of the dogs after the use of the TTA technique and implantation of MSCs at the osteotomy site. Our hypothesis is that the use of MSC will accelerate the cicatricial process after TTA in dogs.

## Methods

A total of 9 male and female dogs from 1 to 12 years old, including Maltese, Cane Corso, Shar-Pei, Labrador, Pitbull, and mixed breeds, were selected in the orthopedic clinical routine of the veterinary hospital Dr. Antônio Clemenceau. All dogs had some degree of claudication and were diagnosed with CCLR through clinical examination, drawer test, and tibial compression, as well as radiographic analysis in specific positioning for TTA according to Hoffmann et al. (2006). The selected animals had complete and unilateral CCLR of the knee joint, with three (33%) showing symptoms in the right knee and six (67%) in the left knee. Five (55%) used cage number 6 mm, three (33%) used the cage number 9 mm, and only one (11%) used the cage number 12 mm. The plates were chosen according to the radiographic pattern with the animals properly positioned (femoro-tibial-patellar joint with 135° angulation) and two plates at number 0, one plate at number 0.5, two plates at number 1, plate number 2, two plates at number 3 and a plate at number 4.

### Surgery procedures

Lateral radiographic projection of the affected limb was obtained pre-operatively to assess the stifle joint. The lateral projection was centered on the stifle joint with superimposition of both femoral condyles. The stifle joint was angled at 135° in accordance to the long axis of the femur. The joint was positioned so that there was no cranial tibial translocation. A standardized TTA transparency (Focus®, Indaiatuba, Brazil) was used over the radiograph to determine the amount of TTA required to position the patellar tendon perpendicular to the tibial plateau.

The dogs were positioned in dorsal recumbency and the affected limb was aseptically prepared. All dogs received ceftriaxone (25 mg/kg) preoperative.

A medial parapatellar incision was performed 2 cm proximally to the tibial plateau and extended distally to the tibial diaphysis. The periosteum of tibial crest was reflected cranially to expose the cranial bone margin of the entire tibial crest. A previously selected TTA bone plate (Focus®, Indaiatuba, Brazil) was positioned over the tibial crest and fixed by 2 or 3 locking screws, depending on the length of the plate. After that, a bicortical osteotomy was performed in the tibial crest observing the plate contour.

The tibial crest, with attached plate, was moved cranially using a spacer and a previously selected cage (Focus®, Indaiatuba, Brazil), was placed into the osteotomy site at the proximal extent of the osteotomy and secured at its caudal margin to the tibia with a 2.0 mm screw. The cranial cage screw was secured into the tibial tuberosity and the plate was then secured distally to the tibia with the appropriately sized screws (2.0 mm; 2.7 mm or 3.5 mm). The surgical site was then sutured routinely.

### Isolation and preparation of MSCs

After the TTA surgery, a subcutaneous fragment of adipose tissue (approximately 20 g) from the medial parapatellar region was collected from all animals. The specimens were stored in 199 medium (M4530, Sigma Aldrich, São Paulo, Brazil) cooled, and sent to the Biocell laboratory (Brasília, Brazil), which specializes in growth and culture of stem cells. There, the adipose tissue fragment was submitted to enzymatic treatment for extraction and expansion of the mesenchymal stem cells following a protocol described by Kang et al. (2012).

**Fig. 1:**
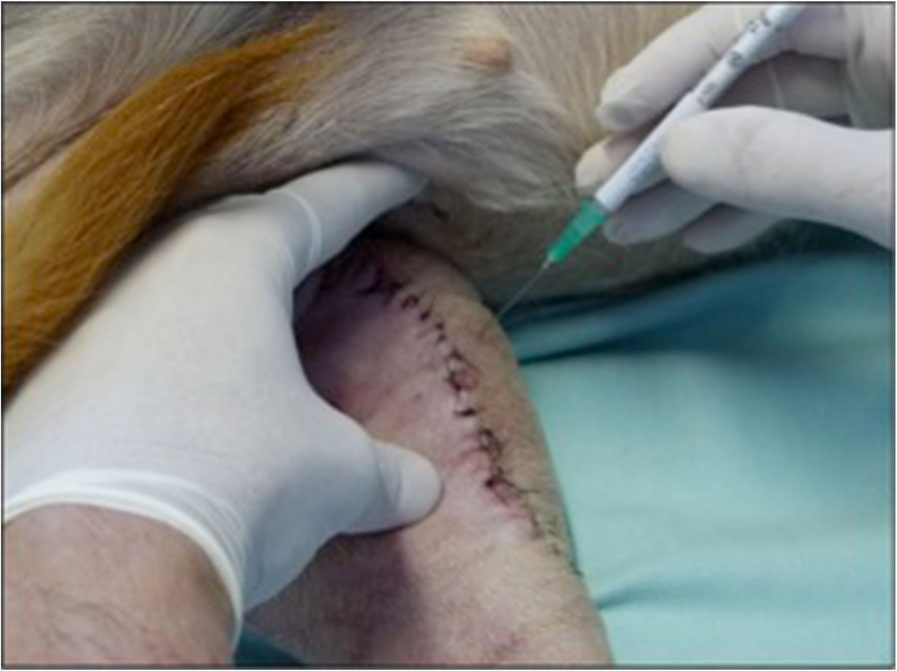
Use of stem cells. Photograph in craniomedial view demonstrating percutaneous application of stem cells at the osteotomy site 8 days after the surgical procedure

The stem cells were maintained in the culture medium (37 °C, 5% of CO2) until the eighth postoperative day, and then about 1.5 million stem cells were removed from the bottles by trypsinization and transferred with transport medium to 1.0-mL hypodermic syringes and applied to the gap formed by the osteotomy. The gap formed by the osteotomy was localized through palpation, and then the cells were injected into the gap percutaneously using a 1 ml syringe with a hypodermic needle (30 × 0.8 mm, Becton Dickinson, São Paulo, Brazil) (Fig. 1).

In the animals of the control group (GC) (*n* = 5), only the culture medium was applied without the MSC. In the animals of the treated group (GT) (*n* = 4), the culture medium was treated with MSC. Only the laboratory was aware of which animals would receive transport with MSC and which received only the transport medium. Thus, the study was characterized as double blind, since neither the surgeon nor the owner knew the composition of groups.

### Qualitative evaluation of radiographic images

On the fifteenth postoperative day, the animals were evaluated for the clinical condition of the surgical wound, degree of claudication, effectiveness of the TTA technique in eliminating the shear movement in the operated knee, and the level of pain in the operated area.

**Fig. 2:**
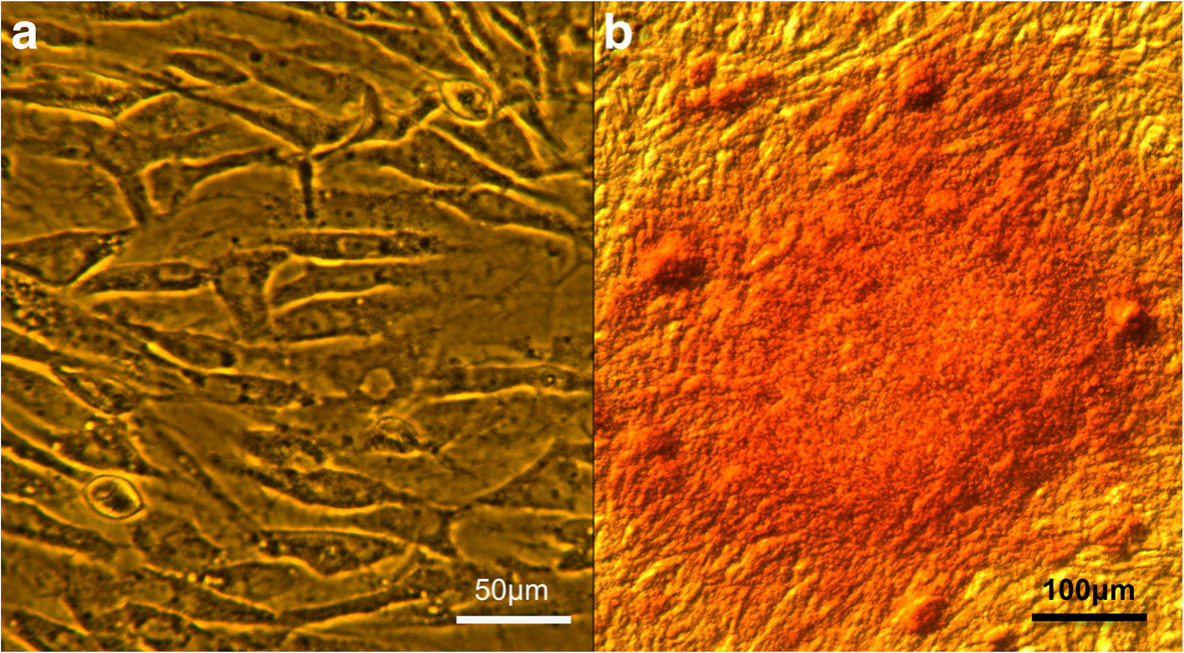
Photomicrograph of stem cells. Photomicrography demonstrating MSC (**a**) and osteocytes after Von Kossa staining demonstrating the calcium (reddish coloration) (**b**)

The animals were X-rayed (Compact Plus 500 L, Philips Medical Systems, São Paulo, Brazil) at 15, 30, 60, 90 and 120 days after surgery. Radiographic images were evaluated by two veterinarians who classified them according to Hoffmann et al. (2006). The classification was made based on the following scores:

- zero - indicates that there was no bone healing;
- I - represents early bone production without bridging the tibial tuberosity and the axis of the tibia;
- II - significant bone bridge in one site;
- III - indicates bone bridge in two sites;
- IV - represents bone bridge at all three sites, including the region of osteotomy proximal to the cage, the region of osteotomy between the cage and the plate, and the distal region of the osteotomy to the plaque (Fig. 2).

### Evaluation of the osteogenic potential of MSCs

To assess the osteogenic potential of MSC culture, some of the cells proliferated in the laboratory were induced to differentiate into bone tissue. For in vitro osteogenic differentiation, the purified MSCs were maintained in a culture medium containing ascorbic acid (50 μM), dexamethasone (0.1 μM) and beta-glycerol phosphate (10 mM) according to the protocol established by Kang et al. (2012). After cell differentiation, the detection of the bone matrix was performed by cytochemical staining with Von Kossa staining (Fig. 2).

Another part of the cells was sent to the MOFA Global laboratory (Verona, WI, USA) to perform the molecular characterization through flow cytometry (AMNIS®, Merck, Darmstadt, Germany) with quantification by image, also evaluating the degree of purity, functionality and viability of such cells, through the identification of specific molecular markers and DNA dyes. For this, 1,000,000 cells were incubated with specific antibodies, capable of identifying proteins that are found only in stem cells, called molecular markers. Such proteins, CD44, CD29, CD90, these proteins are found in the plasma membrane of stem cells with specific functions.

In addition, to analyze the cell population and to quantify the degree of functionality, antibodies were used to identify intracytoplasmic molecular markers, such as the OCT3/4 and SOX2 proteins, responsible for the maintenance of the undifferentiated state of the stem cells. Likewise, cytometry was able to identify the proportion of undifferentiated cells found in the sample.

### Analysis of the density of bone trabeculae in the spongy substance

To evaluate the density of bone trabeculae in the spongy substance, the radiographic images captured at different times were processed in an image editing software (Adobe Photoshop CS6, version 6.0, Adobe Systems Inc., San Jose, CA, USA) to delimit the area of interest in standardized size, according to Mahl et al. (2009).

After the delineation of the cuts representing the spongy substance in the region of the osteotomy site after the surgical procedure, the analyzed images of the groups were processed in Image-Pro Plus 4.1 image analysis software (Media Cybernetics Inc. Silver Springs, MD) according to Blatt et al. (2004).

### Statistical analysis

A descriptive analysis was performed to obtain the mean and standard deviation, followed by the Kolmogorov-Smirnov test. The radiographs were divided into groups in the postoperative evaluation periods so that the images were classified as proposed by Hoffmann et al. (2006) in different scores and different periods from the application of the Chi-square test. The density of the bone trabeculae arranged in the spongy substance was evaluated in each of the periods for the two groups by applying the Mann-Whitney test. The density was also compared between the different periods from the application of Tukey’s multiple comparison tests between the two groups. Statistical significance was set at *p* ≤ 0.05 for all tests, and the Graph Pad Prism 6.0 program was used.

## Results and discussion

**Fig. 3:**
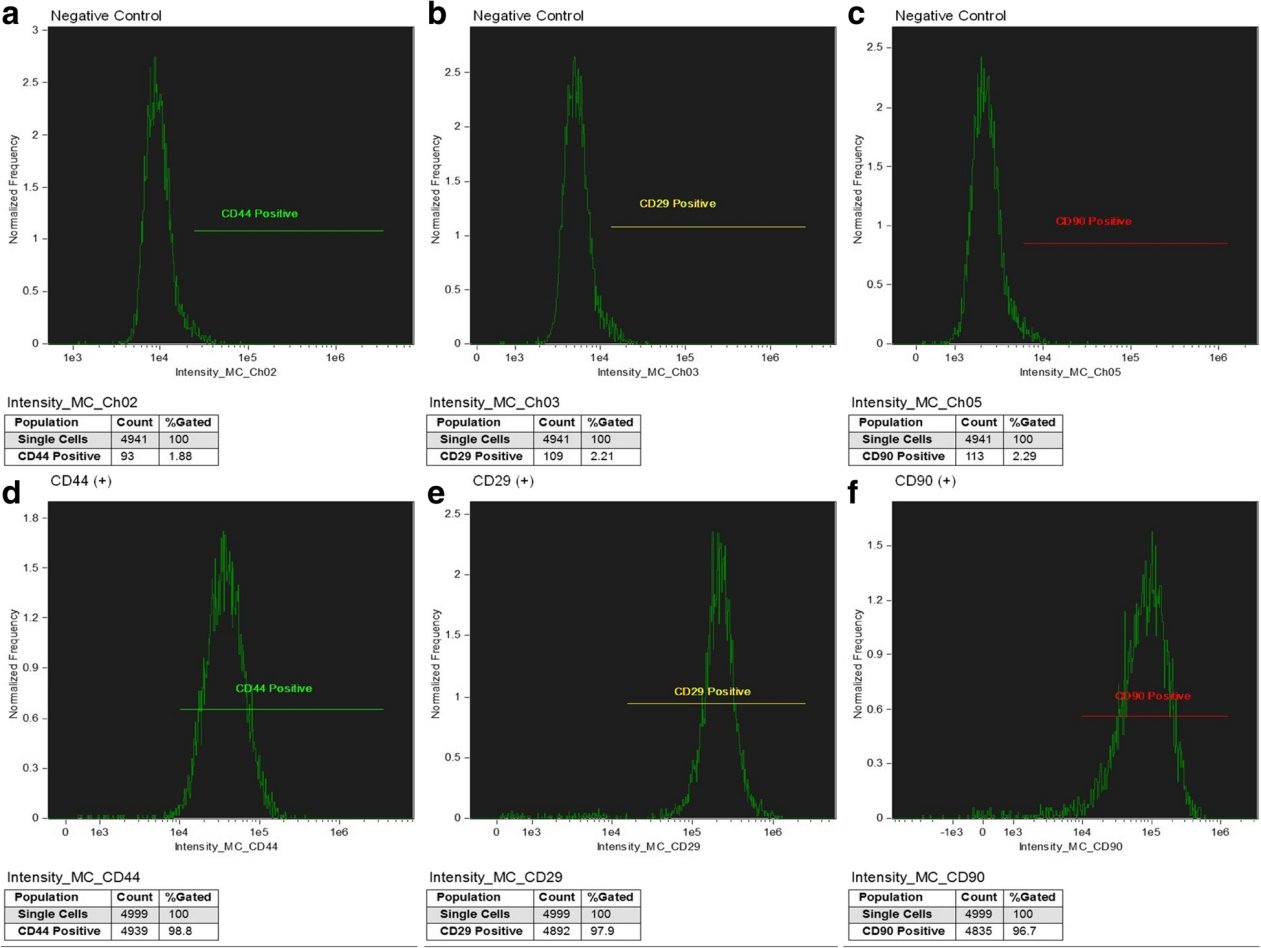
Representative data for the expression of osteocyte mesenchymal stem cell surface markers markers from negative (**a**, **b** and **c**) and positive (**d**, **e** and **f**) controls using immunoglobulin and specific antibody for CD44, CD29 and CD90. The number of cells analyzed was 1,000,000 for each of the labeled cell types. Purchased from AMNIS employing DiVa software 4.0 and analyzed with Flowjo / Mac version 9.4.7

In order to ensure that the isolated cells are mesenchymal stem cells, we used the CD44, CD29 and CD90 markers, which were positive, indicating that the sample consisted of mesenchymal cells (Fig. 3).

**Fig. 4:**
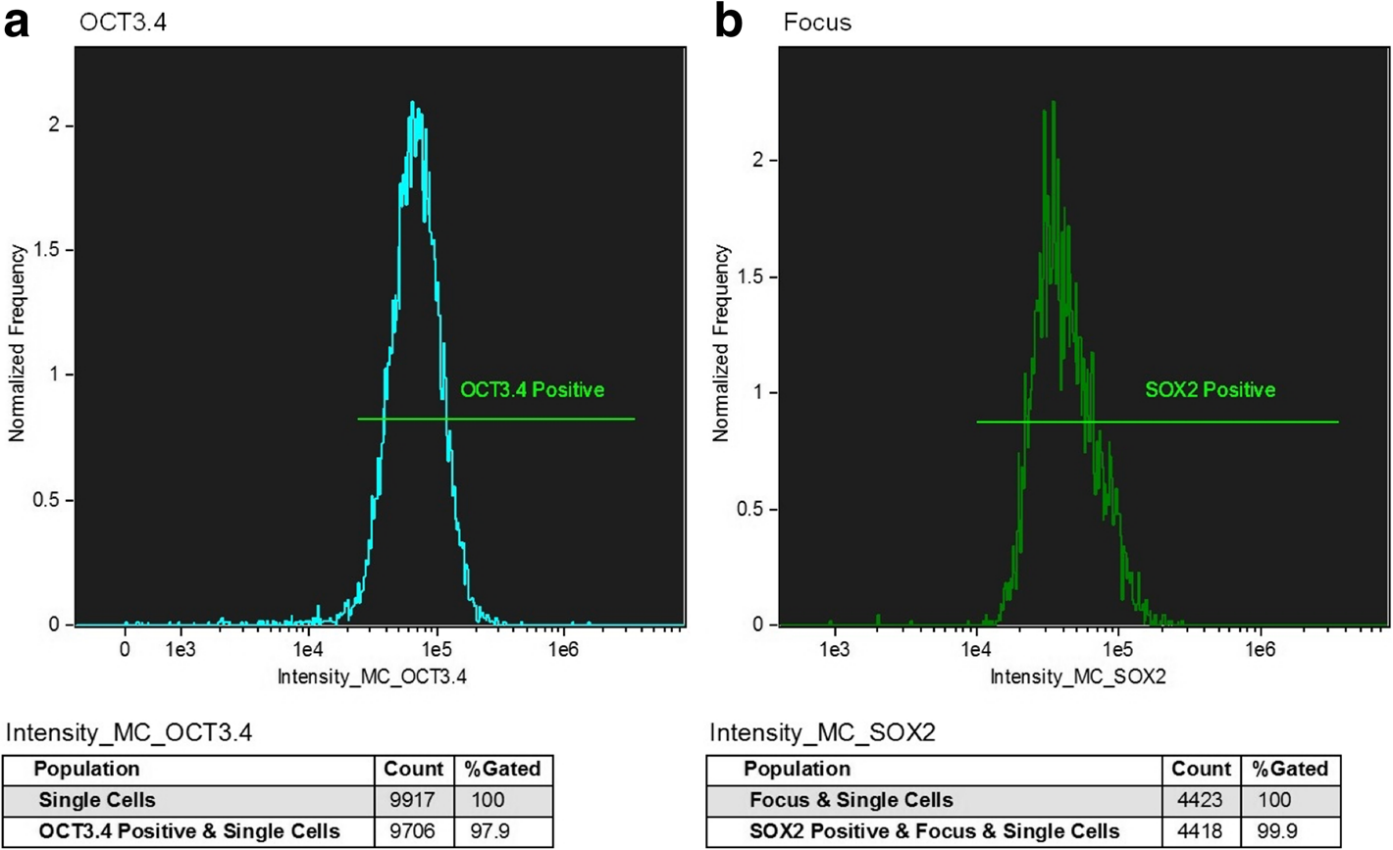
Expression of cell markers OCT3.4 (**a**) and SOX2 (**b**) in mesenchymal stem cells of osteocytes using immunoglobulin and specific antibody. The number of cells analyzed was 1,000,000 for each of the labeled cell types, Purchased from AMNIS employing DiVa software 4.0 and analyzed with Flowjo / Mac version 9.4.7

Through flow cytometry results, we observed that 97.9% of the cells were OCT3/4 positive and 99.9% of the cells were SOX2 positive, thereby ensuring that the cells maintained the undifferentiated state of the stem cells. In addition, the OCT3/4 and SOX2 transcription factor are involved in the maintenance of the proliferative state of the stem cells, it being desirable that such cells present these factors (Fig. 4).

**Fig. 5:**
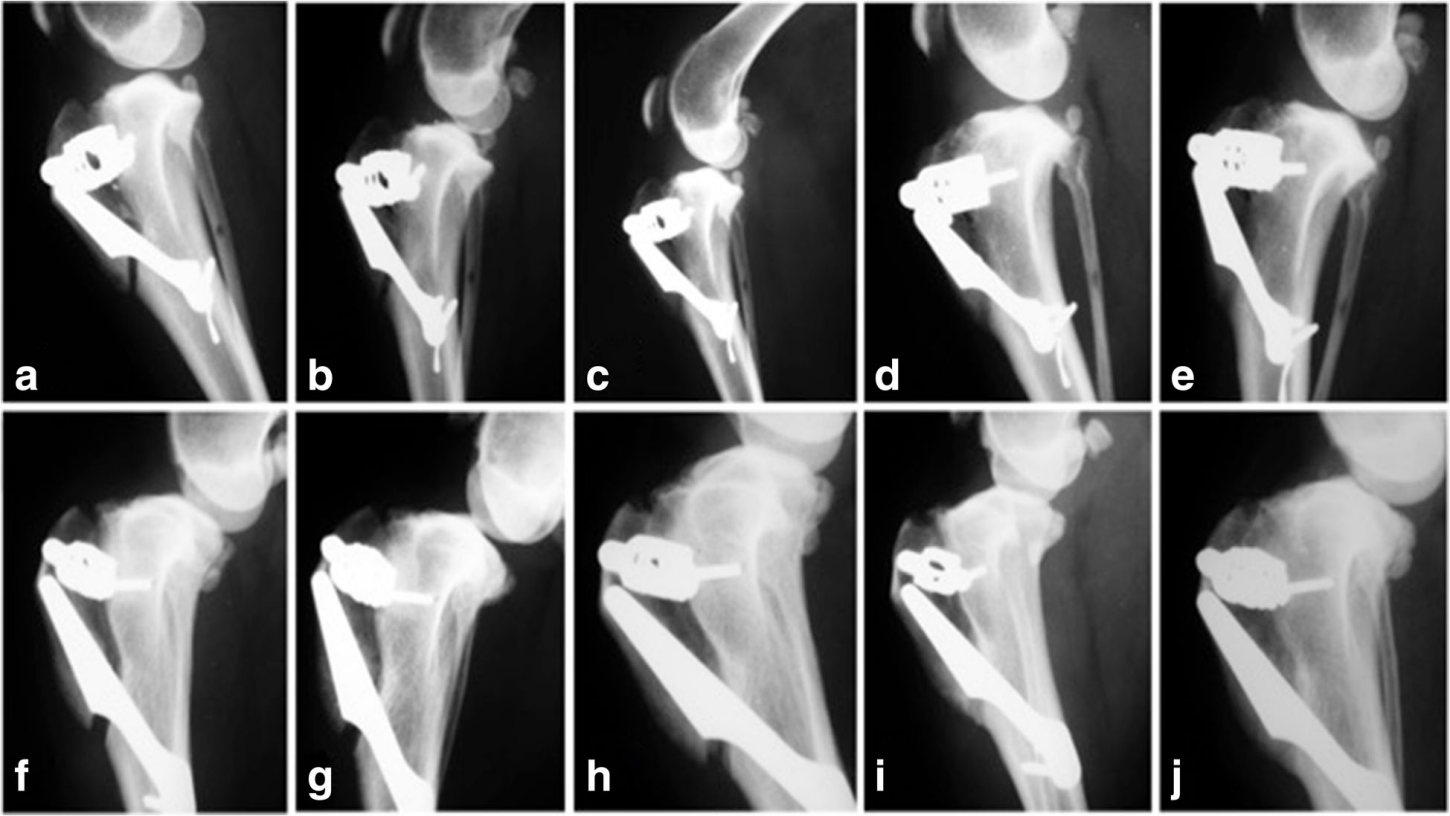
Radiographs showing the periods evaluated and the scores. Images **a**, **b**, **c**, **d** and **e** represent animals of the GT, respectively, in the periods of 15, 30, 60, 90 and 120 days. Images **f**, **g**, **h**, **i** and **j** represent GC animals, respectively, in the periods of 15, 30, 60, 90 and 120 days. Score II (**a**), Score III (**b**, **f** and G), Score IV (**c**, **d**, **e**, **h**, **i** and **j**)

The radiographs of the groups were divided over the postoperative evaluation periods (Fig. 5) so that the images were classified according to the table below, and the scores were evaluated before the different periods according to the application of the Chi-square test (Table 1).

**Table 1:** Classification of ossification (%), according to Hoffman et al. 2006, of the animals from the control group (GC) and those treated with mesenchymal stem cells group (GT) throughout the evaluation period

*SCORE*	Day 15		Day 30		Day 60		Day 90		Day 120		*p*
	GC	GT	GC	GT	GC	GT	GC	GT	GC	GT	
ZERO	50	75	20	50	10	–	–	–	–	–	< 0,0001
I	30	12,5	10	12,5	10	12,5	–	–	10	12,5	0,07
II	–	12,5	40	25	50	25	30	–	20	–	< 0,0001
III	20	–	20	12,5	–	50	30	62,5	30	50	< 0,0001
IV	–	–	10	–	30	12,5	40	37,5	40	37,5	0,009

The Chi-square test with a significance level of *p* ≤ 0.05 was performed between the same periods for the different groups

Comparing the bone trabeculae density of the newly formed material after the osteotomy between the different groups, it was observed that the GC animals achieved a mean ossification 36.45% greater after 30 days compared to the GT (*P* ≤ 0.033). In the other periods, there was no statistically significant difference between the groups (Table 2).

**Table 2:** **-** Mean + standard deviation of the mean of the trabeculae density of the spongy substance (%) in the control (GC) and treated (GT) groups. The Mann-Whitney test was applied between the groups in each period

	Day 15	day 30	Day 60	Day 90	Day 120
GC	39,32 ± 6,42	71,50 ± 4,43*	70,99 ± 3,95	66,40 ± 5,51	66,67 ± 8,26
GT	48,81 ± 7,22	45,44 ± 7,34	64,50 ± 9,78	61,25 ± 4,49	68,45 ± 5,55

Asterisk (*) expressed statistical difference *p* ≤ 0.05 between the control group and the treated group at the same evaluation time. The absence of an asterisk means that there was no statistical difference between the two groups at the same time of evaluation

Relatively and partially, and based only on the assessment of claudication, it was observed that the GT animals had a similar response to GC animals at the end of the study. Thus, it was possible to extrapolate that the application of mesenchymal stem cells was not as beneficial as expected with regard to ambulation and reduction of immediate postoperative discomfort for the treated group. This study did not agree with Hoffmann et al. (2006); Lafaver et al. (2007), in which the evaluated animals had an effective response even in the short term.

In both objective and subjective evaluations, it was observed that the GC obtained results were equal to the GT in relation to the ossification process at the end of the experiment. However, the GC may have obtained a delay in the period of differentiation, which can be justified due to the necessity of reorganization due to the inflammatory process after the postoperative introduction of the stem cells eight days later, unlike the study by Zamprogno (2007), which found efficacy in therapy with stem cells introduced after 15 days of the first intervention in fractures and promoted bone union by the end of the study.

As a result, the application of cells should perhaps have occurred earlier, since the process of cell differentiation should have started from the moment of their application. Contrary to this assessment, the cells applied were probably diverted, in part, due to the reorganization of the inflammatory process present after their introduction into the medium. Taking into account that the first inflammatory stage occurs from the first day of injury to the third day (Cho et al. 2002) and that during this period, the cleaning of damaged areas and migration of pluripotent cells are performed, immunosuppressive functions and reduction of the immune cell response are activated (El-Jawhari et al. 2016), thus justifying the observed response.

In the second phase, called the repair or chondrogenic phase and characterized by the peak of collagen expression from the seventh to the fourteenth day (Cho et al. 2002), differentiation of pluripotent cells into osteoblasts or chondroblasts was observed. This phenomenon was induced by macrophages, which also regulated the differentiation of pluripotent cells and thus the action of other immune cells, including T and B lymphocytes located near the osteoblasts, thus aiding in the differentiation of cartilage and bone (El-Jawhari et al. 2016).

Bone fracture promotes disruption of blood supply and subsequent platelet aggregation with the release of proinflammatory cytokines. These cytokines stimulate the return of lymphocytes and monocytes/macrophages to the site of the fracture, initiating the process of bone healing (El-Jawhari et al. 2016).

The third and final phase, called osteogenic or remodeling, begins in the first 24 h after the injury, and the maximum differentiation occurs between days 14 and 21 after the fracture (Cho et al. 2002). Thus, in the two GT cases where there was a fracture at 15 days, this may have influenced the observed healing delay in relation to GC animals that presented the fracture late, suggesting that the introduced stem cells may not have differentiated into osteoblasts, as expected, but again diverted to the new inflammatory process installed after the fracture of the tibial tuberosity. In addition, there is the fact that the timing of the therapeutic intervention in relation to the inflammatory state is an essential challenge to be addressed according to El-Jawhari et al. (2016). In any case, both groups achieved complete ossification within the postoperative period of 8 to 10 weeks, as proposed by Hoffmann et al. (2006).

Although there was complete ossification within the estimated period, the therapeutic use of MSC for bone loss recovery did not achieve statistically significant results as expected in relation to minimizing the estimated gap recovery time. The microenvironment of the lesion, and particularly the local inflammation, interfered with the action of the stem cells applied in the region of the osteotomy.

## Conclusions

The results obtained in this study were not those expected by the hypothesis we outlined at the beginning, but we must be careful not to jump to conclusions. This study used a great variability of ages and dog breeds. Perhaps we would have obtained a different result if the sample was more homogeneous, but the sample used in this study represents the variability that we verified in the clinical routine. In the future, we intend to carry out new research in order to overcome these limitations. However, we believe that we use a very important technique in the routine, so these results, even if partially inconclusive, should be shown to delineate research and future uses in the clinic.

## Abbreviations

CCLR: cranial cruciate ligament rupture
GC: control group
GT: treated group
MSC: mesenchymal stem cells
TTA: tibial tuberosity advancement
